# Serotype Diversity of Astroviruses in Rawalpindi, Pakistan during 2009–2010

**DOI:** 10.1371/journal.pone.0061667

**Published:** 2013-04-18

**Authors:** Muhammad Masroor Alam, Adnan Khurshid, Muhammad Suleman Rana, Shahzad Shaukat, Salmaan Sharif, Mehar Angez, Muhammad Naeem, Syed Sohail Zahoor Zaidi

**Affiliations:** 1 Department of Biotechnology, Quaid-i-Azam University, Islamabad, Pakistan; 2 Department of Virology, National Institute of Health, Islamabad, Pakistan; National Taiwan University Hospital, Taiwan

## Abstract

Astroviruses are globally known enteropathogens causing gastroenteritis and diarrhea, with eight well defined serotypes. Epidemiological studies have recognized serotype-1 as the most common subtype but no such data is available in Pakistan. During 2009–2010, we found astroviruses in 41 out of 535 (7%) samples collected from hospitalized children. Thirty one strains belonged to serotype-1 and clustered into two distinct lineages. Serotype-3, -4 and -6 were detected with 97–98% genetic homology to Indian and Chinese strains.

## Introduction

Astroviruses have been known as the causative agents of gastroenteritis since 1975 [1]. The virion particles are non-enveloped, containing positive sense single stranded RNA of 6.8 to 7.2 kb in length enclosed in a nucleocapsid coat [2]. Three open reading frames have been identified; ORF1a and 1b encoding protease and polymerase proteins respectively while ORF2 encodes the capsid protein precursor [3], [4]. ORF1b is expressed as a fusion protein generated through a ribosomal frameshift mechanism using a highly conserved heptameric slippery sequence between ORF1a and 1b [5], [6]. These viruses comprise 2 genera, *Mamastroviruses* and *Avastroviruses* within the family *Astroviridae* infecting mammalian and avian species respectively [7]. The astrovirus taxonomy is mainly based on the species of origin and the serotypes within each species are defined on the basis of twenty-fold or greater cross-neutralization titers. The poor adaptation of astroviruses to laboratory growth systems led to the formation of genome based classification, based on the percentage similarity of nucleotide and amino acid sequence of ORF2 capsid protein [8]. In humans, eight classic serotypes of astroviruses are known (HAstV1-8) while five new species have been identified recently named as AstV-MLB-1 & -2 (Finkbeiner SR. Virol J. 2008 and Finkbeiner SR. Virol J. 2009), AstV-VA1 (Finkbeiner J Virol 2009), AstV-HMO-A (Kapoor A. JGV 2009) and AstV-HMO-B (Kapoor A. JGV 2009).

Human astroviruses mainly infect children (<2 years of age) along with adults as well as immunocompromised persons and are found to cause 20% of the sporadic non-bacterial diarrhea cases [9]. The prevalence rate varies from 10–30% depending upon the geographical region with 33–65% of mixed infections with rotavirus and norovirus [10], [11].

## Materials and Methods

**Figure 1:**
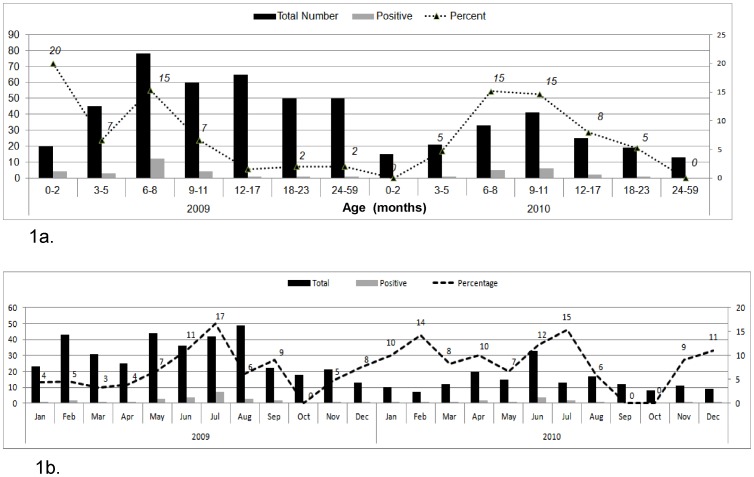
Astrovirus detection rates in various age groups of children hospitalized during 2009–2010. The data labels indicate the percentage of positive samples among each age group (1a). Month wise distribution of astrovirus positive samples between January, 2009 to December, 2010 (1b).

This study was approved from the National Institute of Health internal review board committee. To investigate the role of astroviruses in childhood diarrhea and its contributing serotypes, we analyzed stool samples of children under 5 years of age who were admitted to a local hospital in Rawalpindi district of Pakistan. Samples from hospitalized children suspected of gastroenteritis were collected as per World Health Organization's standard case definitions that describe a suspected case if a child is reported with three or more loose stools or any vomiting during 24 hours. 535 samples were collected over a period of two years (2009–2010) with their epidemiological details as given in Figure 1. The samples were spun to get clarified fecal supernatant and frozen at −20°C until further analysis. RNA was purified using QIAamp viral RNA minikit (Qiagen Inc., USA) and kept at −80°C. Reverse-transcription PCR to detect astrovirus nucleic acid was performed using the ORF2 targeted primers, Mon-269/Mon-270, as described previously [12]. The amplified product was purified and bidirectionally sequenced using BigDye terminator v3.1 cycle sequencing kit (Applied Biosystems, USA). The raw sequence reads generated by automated Genetic Analyzer 3130xl were edited and refined to get a single consensus read using the program Sequencher version 5.0 (Gene Codes Corporation, Ann Arbor, MI USA). Nucleotide alignments and phylogenetic analysis were conducted using MEGA 4.0.

## Results

Out of total 535 (368, 2009; 167, 2010) samples analyzed for astrovirus, 7.6% (n = 41) were found positive for astrovirus RNA with 7% (n = 26) and 9% (n = 15) during 2009 and 2010 respectively. Higher proportion of astrovirus infections was found in children between 3–12 months of age as compared to older children (85% and 15% respectively), while no significant difference was found between males and females (Figure 1a and 1b). Co-infection with rotavirus and norovirus was found in 3% (16 out of 535) and 1% (5 out of 535) subjects respectively.

**Figure 2:**
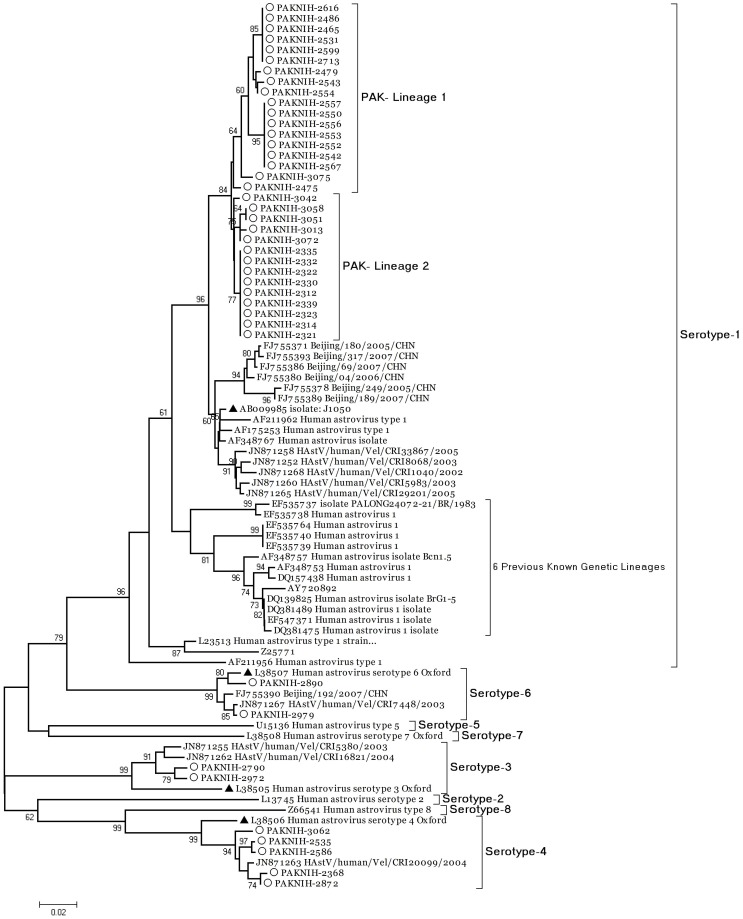
Phylogenetic analysis of Human Astrovirus strains identified in this study based on the partial ORF2 region (348 bases) encoding outer capsid precursor protein. The reference strains and closest match isolates detected through BLAST are given for genetic comparison. The phylogenetic tree with 500 bootstrap replicates was reconstructed using neighbor joining method and the K-2P model through MEGA 4.0. Serotype-1 strains were compared to four prototype strains (USA-GenBank accession number L23513, isolated in 1994), United Kingdom (GenBank accession number Z25771, isolated in 1990), Japan (GenBank accession number AB009985, isolated in 1997) and Germany (GenBank accession number AY720892). Taxa with arrow head indicates prototype strains within each serotype.

Molecular analysis revealed that the astroviruses found in Rawalpindi district belong to four different types HAstV-1, -3, -4 and -6 with 78%, 5%, 12% and 5% isolates respectively. Type-1, -3, -4 were detected in both years while serotype-6 was detected in two samples during 2010 only. Thirty two serotype-1 strains identified in this study had an inter-strain nucleotide similarity of 98–100% within the 348 bases of ORF2 region sequenced. Online BLAST search (www.ncbi.nlm.nih.gov/blast) revealed the closest nucleotide identity of all Pakistani astrovirus strains with those from the neighboring countries i.e. China and India. These closest matched type-1 viruses when compared and phylogeneticaly analyzed, presented ∼4–5% nucleotide differences with Indian and Chinese strains and were grouped into two distinct lineages in the ORF2 based tree (Figure 2). In contrast, all of the viruses from serotypes-3, -4 and -6 were found clustered among the strains from India. The closest match for serotype-3 strains was Indian strain reported from Velore district in 2003 and 2004 (GenBank accession number JN871255 and JN871262). Similarly, the serotype-4 viruses were phylogeneticaly positioned among Indian strains found during 2004 (GenBank accession number JN871263). The serotype-6 strains from Pakistan presented two separate lineages, PAKNIH-2890 grouped with prototype strain (GenBank accession number L38507, isolated in UK) while the second lineage contained closely matched viruses from Beijing, China (GenBank accession number FJ755390) and Velore, India (GenBank accession number JN871267).

In addition, the Pakistani viruses detected in this study were also compared to sequences of prototype strains available in GenBank. For serotype-1 astroviruses, the nucleotide sequence of 348 bases from ORF2 region was aligned and compared to the four prototype strains reported from USA, United Kingdom, Japan and Germany. The closest identity of Pakistani strains was found with Japanese prototype strain showing 96–98% nucleotide similarity while the other prototype strains showed a greater degree of variance e.g. 91–93%, 88–89% and 88–91% divergence against USA, UK and Germany type 1 strains respectively. Significant nucleotide variance of 7% was found among serotype-3 viruses and its prototype (GenBank Accession No. L38505, Oxford strain). For serotype-4 and -6, the nucleotide variation was 5% and 1–2% with the prototype strains respectively (GenBank Accession No. L38506 and L38507).

## Discussion

Unlike developed countries, epidemiological studies have never been conducted in Pakistan to investigate the burden of viruses in gastroenteritis and diarrhea related infections. Although a national program of laboratory based rotavirus surveillance was initiated in 2006, this program is based on the antigenic detection of rotavirus in stool samples. Similarly, no further investigation is routinely made at public health facilities for viral etiologies of diarrhea or gastroenteritis. Pakistan has been known to be among the top five countries with highest number of reported childhood deaths due to diarrhea. To the best of our knowledge, we are, hereby, first to report the molecular epidemiology and genetic characterization of astroviruses in children admitted to a local tertiary care hospital in Pakistan. The only previous study in the country was conducted approximately two decades ago on samples collected during 1990–1994 in Karachi district and reported 11% samples positive for astrovirus type 1 [13]. Recently, novel astroviruses designated as AstV-HMO-A and AstV-HMO-B have been identified in Pakistan and Nigeria through metagenomic analysis of pediatric stool samples [14] but none of these new variants have been found in the present study which can be attributed to our use of highly specific primers targeting only the classical astrovirus serotypes. Upon nucleotide comparison of Mon-269/Mon-270 primers used in this study with the target region of ORF-2 (4526–4974 nucleotides based on astrovirus type 1; Genbank accession No. Z25771), several variations were found among the recently discovered astrovirus species necessitating the use of broad-range primers that can also detect the new astrovius species like AstV-MLB-1 and -2, AstV-HMO-A (highly similar to AstV-VA2), AstV-HMO-B (highly similar to AstV-VA3), and AstV-VA1 (highly similar to AstV-HMO-C) (Finkbeiner SR. Virol J. 2008 and Finkbeiner SR. Virol J. 2009, Finkbeiner J Virol 2009, Kapoor A. JGV 2009).

Our findings substantiate the data of epidemiological surveys in different countries that serotype-1 prevails as the most common subtypes while serotype-2, 3, 4 and 5 are less commonly detected, in addition to 6, 7 and 8 which are rarely found [15]–[17]. The current data represents the cases that were severely infected and required hospitalization but there may be the possibility of other serotypes prevailing in our population causing less severe or asymptomatic disease as reported previously from Australia [18]. The astrovirus strains identified in Rawalpindi during the two consecutive years period (2009–2010) does not represent any change in deduced amino acid sequence as compared to the prototype strains representing a stringent stability of the ORF2 region of virus genome. Similar findings have been observed by Mustafa et al.; Palombo et al., and Schnagl et al., where strains collected over 17 years period from Australia did not show any amino acid change in the same region of ORF2 encoded capsid protein [17]–[19]. Although the same genomic region has been utilized to identify the genotypic information [15], [17], [19], [20] but this part of ORF2 does not provide sufficient information for evolutionary studies due to its high level of conservation and needs inclusion of other genomic regions. The serotype-1 astroviruses detected in our samples constitute a separate cluster and might reflect a different ancestral origin compared to those from neighboring countries. Recently, six lineages (1a–1f) have been proposed for astrovirus serotype-1 strains based on the 348 nucleotide region of ORF2 [21] but our viruses do not cluster with any of these lineages and thus support the circulation of type-1 strains with sufficient genetic variability as compared to the earlier known strains. Similar findings of geographical-specific clustering has been reported by Noel at al. [12] for type 1 viruses collected from different regions like Australia, New York and Colorado. Such findings also emphasize the role of continuous monitoring through large scale surveys to better understand the genetic diversity and astrovirus epidemiology in a particular population.

## Conclusion

In Conclusion, Rawalpindi, one of the most populated city of Punjab province, is the only district for which more recent epidemiological information has been generated for astrovirus and its contributing serotypes. Further, Pakistani human population harbors a wide variety of astrovirus strains that require evaluation through large-scale incessant epidemiological surveys. As highlighted by Liu et al., such studies are helpful for formulating a more universal vaccine especially against serotype-1 which is found at significantly higher rates across the globe [20].
